# USA300 Methicillin-Resistant *Staphylococcus aureus*, United States, 2000–2013

**DOI:** 10.3201/eid2111.150452

**Published:** 2015-11

**Authors:** Margaret Carrel, Eli N. Perencevich, Michael Z. David

**Affiliations:** University of Iowa, Iowa City, Iowa, USA (M. Carrel, E.N. Perencevich);; Iowa Veterans Administration Health Care System, Iowa City (E.N. Perencevich);; University of Chicago, Chicago, Illinois, USA (M.Z. David)

**Keywords:** Methicillin-resistant Staphylococcus aureus, MRSA, USA300, spatiotemporal analysis, systematic review, bacteria, antimicrobial resistance, United States

## Abstract

In the United States, methicillin-resistant *Staphylococcus aureus* (MRSA) with the USA300 pulsed-field gel electrophoresis type causes most community-associated MRSA infections and is an increasingly common cause of health care–associated MRSA infections. USA300 probably emerged during the early 1990s. To assess the spatiotemporal diffusion of USA300 MRSA and USA100 MRSA throughout the United States, we systematically reviewed 354 articles for data on 33,543 isolates, of which 8,092 were classified as USA300 and 2,595 as USA100. Using the biomedical literature as a proxy for USA300 prevalence among genotyped MRSA samples, we found that USA300 was isolated during 2000 in several states, including California, Texas, and midwestern states. The geographic mean center of USA300 MRSA then shifted eastward from 2000 to 2013. Analyzing genotyping studies enabled us to track the emergence of a new, successful MRSA type in space and time across the country.

## Abstract 2

We confirm USA300 in the West and Midwest and subsequent diffusion to the East Coast.

*Staphylococcus aureus* is among the most common causes of bacterial infections in humans and probably has been a member of the human commensal flora for millennia (*1*). Chambers and Deleo identified serial “waves of resistance” in the history of 20th-century *S. aureus* epidemiology (*2*). They described the emergence of penicillin-resistant *S. aureus* in the 1940s and rapid spread during the 1950s and 1960s, initially in the health care setting and then in the community, as the first wave of resistance. With the introduction of semisynthetic antistaphylococcal penicillins, the first of which was methicillin in 1959, the second wave of resistance emerged with methicillin-resistant *S. aureus* (MRSA). Many of the early, or archaic, MRSA clones were related to the so-called “First MRSA” strain, which was later designated as sequence type (ST) 250 by multilocus sequence typing (MLST). These archaic MRSA clones caused health care–associated infections primarily in Europe until the 1980s. At that time, new strains of MRSA predominantly belonging to 5 clonal clusters (CC) (designated with MLST as CC8, CC22, CC5, CC45, and CC30) emerged worldwide, causing the third wave of resistance in *S. aureus* that continued into the 21st century (*2*).

Beginning in the late 1990s, new strain types of non–multidrug-resistant MRSA began to circulate outside the health care setting in the United States, a phenomenon seen even earlier in Australia (*3*). These community-associated MRSA infections, particularly skin and soft tissue infections, became common in the United States after 2000 (*4*). They constituted the fourth wave of resistance for *S. aureus*.

The community-associated MRSA strain USA300, which nearly always carries genes for the Panton-Valentine leukocidin (PVL) and the staphylococcal cassette chromosome *mec* (SCC*mec*) type IV, became the predominant strain type of MRSA circulating in the United States by 2011 (*5*). Community-associated MRSA infections, defined as infections in patients who lacked recent exposure to the health care setting, disproportionately affected children (*6*–*8*), incarcerated populations (*9*–*11*), underserved urban populations (*3*,*12*), and other specific groups (*13*–*17*). Early US reports on community-associated MRSA infections were published from Houston (*18*), Chicago (*7*,*19*) and elsewhere in the Midwest (*20*), Minnesota (*21*), Tennessee (*22*), Hawaii (*23*), and California (*9*,*11*). Soon after it began spreading in the community, USA300 became a common cause of infections in the health care setting as well, blurring the epidemiologic distinction between community-associated and health care–associated MRSA (*24*).

No national surveillance program exists that tracks the incidence of community-associated MRSA infections or the molecular epidemiology of MRSA infections more generally. However, anecdotally community-associated MRSA infections were less common on the US East Coast during the early part of the first decade of the 21st century. Single-center studies on the molecular epidemiology of MRSA isolates causing infections in the country became increasingly common after 1999 as new genotyping schemata were developed and as the price decreased for DNA sequencing. These included typing systems for pulsed-field gel electrophoresis (PFGE) by the Centers for Disease Control and Prevention (*25*), MLST (*26*), and *spa* typing (*27*).

Using the data available from the literature as a proxy for surveillance of USA300 as a proportion of circulating MRSA isolates, we set out to estimate the geographic spread of USA300 from initial reports of infections during 2000–2013 and compare it to the distribution of USA100 MRSA during that time. We compared USA300 to USA100 because they are the predominant MRSA strain types in the United States, and each is typically associated with different acquisition environments. USA300 is most often community associated, whereas USA100 is usually health care associated (*28*). The availability of extensive genotyping studies during the fourth wave of resistance enabled us to track the emergence of a new, successful strain type in space and time during a 14-year period.

## Methods

### Literature Review

We systematically reviewed the literature to identify all peer-reviewed publications, including genotyping information, on MRSA isolates. A PubMed search was conducted for citations related to MRSA published during 2000–2014 by using the following search criteria: (“2000/01/01”[date–publication]: “2014/04/01”[date–publication]) AND ((((((MRSA) OR ORSA) OR methicillin-resistant staphylococcus aureus) OR oxacillin-resistant staphylococcus aureus) OR methicillin-resistant staphylococcus aureus)). This search identified 18,615 possible articles for inclusion. A physician (M.Z.D.) reviewed the titles and abstracts of these 18,615 citations; citations were chosen for a review of the full text if we found evidence that genotyping was performed for the study. Genotyping modalities included in this assessment were MLST, PFGE if a reproducible and well-recognized system of nomenclature was used, *spa* typing, or direct repeat unit typing. This assessment resulted in the full-text evaluation of 3,389 articles published worldwide for genotyping information. These studies were then sorted by the country or countries from which reported MRSA isolates were collected. We excluded from further analysis studies with genotyping information about isolates exclusively from countries other than the United States, and all studies that included genotyping information about at least 1 isolate from the United States were further analyzed. We screened the references cited in the selected articles for additional publications, which were evaluated in full text to assess for inclusion in the study. To avoid duplication, we excluded from consideration studies that included only previously published isolates.

### Data Abstraction

The search criteria identified isolates from 354 articles for inclusion. Data on 33,543 isolates were then abstracted into an Excel database (Microsoft, Redmond, WA, USA), hereafter called the MRSA TypeCat (an abbreviation of the MRSA Typing Catalogue). For each isolate, the geographic place of collection (city, state, or region of the country), year(s) of collection, source of the culture (specific animal species, human, or fomite), and any unique isolate identifier were recorded. Each isolate obtained from humans was recorded if it was obtained from a site of infection or from a culture assessing for asymptomatic colonization, if this information was available. The MRSA isolate was also recorded for human isolates obtained from a site of infection if we considered the infection to be a community-associated or health care–associated MRSA infection by clinical or epidemiologic criteria. Bibliographic information for each article was recorded, including the first author, the journal, year of publication, journal volume, page numbers, and PubMed unique identifier.

For each isolate, the following genotyping information was recorded if it was provided in the article: SCC*mec* type (with citation for the method used to determine SCC*mec* type); MLST type; *spa* type; coagulase type; direct repeat unit type; *agr* type; PFGE type; capsule type; the presence or absence of PVL; and the presence or absence of a marker for the arginine catabolic mobile element, which is frequently present in USA300 MRSA. Information was available for different combinations of typing schemes (Table).

**Table:** Types of genotyping* data in the MRSA TypeCat from isolates, indicating number of isolates with data for each genotyping system or result, United States 2000–2013

Data	No. (%), N = 33,543
MLST	7,104 (21.2)
*spa* typing	7,466 (22.3)
PFGE (USA or USA-like)	22,846 (68.1)
dru Typing	78 (0.23)
SCC*mec* type	13,667 (40.7)
PVL PCR (total tested)	10,782 (32.0)
PVL PCR (positive)	7,370 (22.0)
ACME tested	2,393 (7.68)
Capsule type	70 (0.21)
Coagulase type	48 (0.14)
*agr* type	476 (1.42)

*Because MRSA isolates could have been subjected to >1 typing method, these categories are not mutually exclusive. ACME, arginine catabolic mobile element; dru, direct repeat unit; MLST, multilocus sequence typing; MRSA, methicillin-resistant *Staphylococcus aureus*; PFGE, pulsed-field gel electrophoresis; PVL, Panton-Valentine leukocidin; SCC, staphylococcal cassette chromosome.

### Definitions

We defined USA300 as any isolate that was considered USA300 by PFGE or any isolate that was 1) PVL positive and 2) either *spa* type t008 or MLST type ST8. These last 2 criteria have an approximate specificity for USA300 of 95% and 98%, respectively (*29*). We defined USA100 as any isolate identified as USA100 by PFGE or any isolate with 1) SCC*mec* type II and 2) either *spa* type t002 or MSLT type ST5.

**Figure 1:**
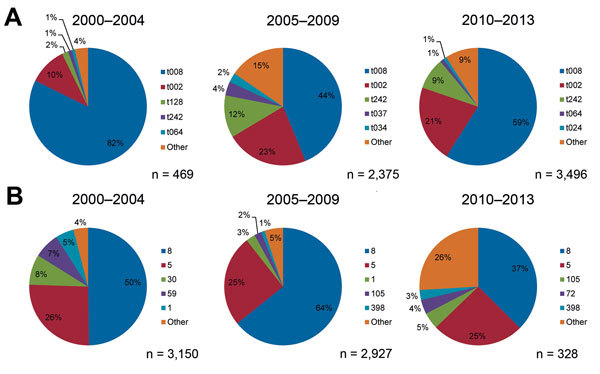
The most frequently reported *spa* types (A) and multilocus sequence types (B) for methicillin-resistant *Staphylococcus aureus* isolates obtained in 2000–2004, 2005–2009, and 2010–2013, United States.

For isolates obtained during a reported period spanning multiple years, the mean year of a study was calculated and was used as the year of collection. If an abstracted published study spanned an even number of years, the mean year was rounded up so that all isolates were assigned an integer year of collection. Eighty-two of the 354 analyzed published studies did not provide dates of collection for the reported MRSA isolates or had assigned years of collection before 2000; we discarded these from further analysis (2,289 isolates). Using these year assignments, we found the 5 most common Ridom *spa* types (*27*) and the 5 most common STs of isolates obtained during the 2000–2004, 2005–2009, and 2010–2013 periods (Figure 1).

### Geographic Analysis

Studies were geocoded to the state in which the reported MRSA isolates were collected. We discarded 46 studies that used data from multiple states (i.e., in which no single US state of collection was reported for specific isolates) or that indicated only regional classifications (11,779 isolates). For each state in each study year, we calculated a proportion of isolates that were defined as either USA300 or USA100 to account for variable sample sizes in states over years.

We generated thematic maps of state-level USA300 and USA100 proportions for 2000–2013 to provide a sense of how these types varied spatially and temporally within the dataset. To visualize simultaneously USA300 and USA100 proportions, as well as the presence of non-USA300 or USA100 MRSA isolates, we created pie charts of these 3 categories for each state during each year.

To determine how the detection of USA300 varied over space and time, we calculated a weighted mean geographic center for 2-year nonoverlapping time increments for USA300 proportions (e.g., 2000–2001, 2002–2003). The weighted geographic mean center for each period is influenced by the states reporting MRSA during that time (influencing the starting location of the geographic center) and by the proportion of isolates defined as USA300 (how states are weighted in the calculation of the center).

### Statistical Analysis

States were assigned to 1 of 4 US Census geographic regions (Northeast, South, Midwest, or West) to test for regional changes in USA300 proportion over time (*30*). Line graphs with percentage of USA300 and USA100 isolates in each year were generated, and a linear regression line was fit for each strain type. A Cochran-Armitage test for trend was implemented in SAS (SAS Institute, Inc., Cary, NC, USA) to assess whether the proportion of isolates in each of the 4 regions defined as USA300 or USA100, respectively, increased during the study period (2000–2013).

## Results

Review of the identified 354 articles that included typed MRSA isolates with genotyping data identified 236 studies that included unique years of bacterial isolation and specified the US states where they were isolated. Within these 236 studies, 19,748 MRSA isolates were reported, of which 8,092 were classified as USA300 and 2,595 as USA100. Among isolates with any reported anatomic site of isolation, skin and soft tissue infections accounted for 62.6% of USA300 and 19.1% of USA100 isolates in studies that reported specific years and geographic locations and 58.8% of USA300 and 7.0% of USA100 in all studies, inclusive of those not reporting state locations or study years. Of all the USA300 and USA100 isolates in spatiotemporal analyses that reported a site of isolation, 38.5% of USA300 and 80.9% of USA100 were known invasive infections, whereas the full 33,543-isolate dataset designated 41.7% of USA300 and 93.0% of USA100 isolates as invasive infections.

Of isolates with reported *spa* types under the Ridom typing system, t008 and t002 were the dominant *spa* types during 2000–2004, 2005–2009, and 2010–2013 (Figure 1, panel A). Although t008 isolates made up the largest percentage during all 3 time groups, the share of isolates with this type decreased from 82% during the first period to 59% during the third, even as the total number of isolates with reported *spa* types increased. The share of isolates categorized as t002 rose from 10% during the first period to ≈20% during the 2 subsequent periods. ST8 and ST5 were the 2 most common MLST types reported during all 3 periods (Figure 1, panel B), as the number of isolates with reported MLST results declined during the study.

**Figure 2:**
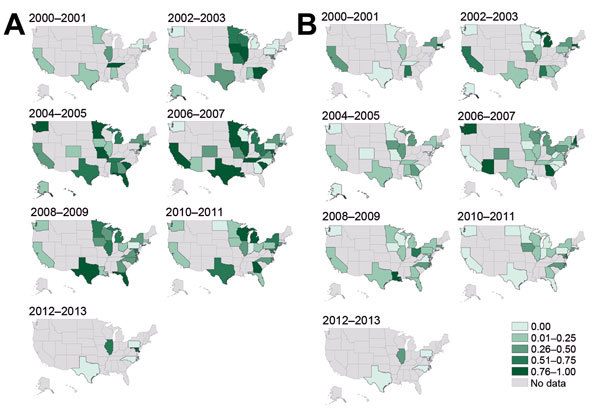
Proportions of methicillin-resistant *Staphylococcus aureus* isolates, United States 2000–2013. A) USA300 strain type. B) USA100 strain type. Darker shading indicates higher proportions of types reported in studies conducted during those years.

The proportion of MRSA isolates defined as USA300 increased during the study period and increased in some states earlier than others (Figure 2). Simultaneously, the proportion of isolates defined as USA100 decreased in many states.

**Figure 3:**
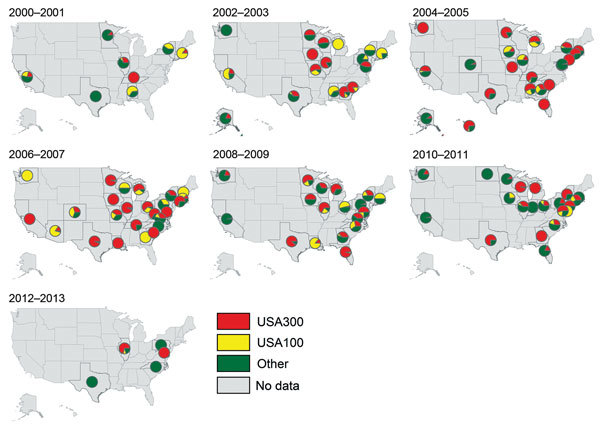
Proportions of methicillin-resistant *Staphylococcus aureus* isolates in each state that were defined as USA300, USA100, or other strain types, United States 2000–2013.

Although USA100 isolates were reported during all years of the study period, pie charts indicate a gradual increase in the proportion of all isolates that were USA300 over time and space (Figure 3; Technical Appendix). In addition, the proportion of studies reporting USA100 simultaneously declined, and a higher percentage of studies reported other types of MRSA.

**Figure 4:**
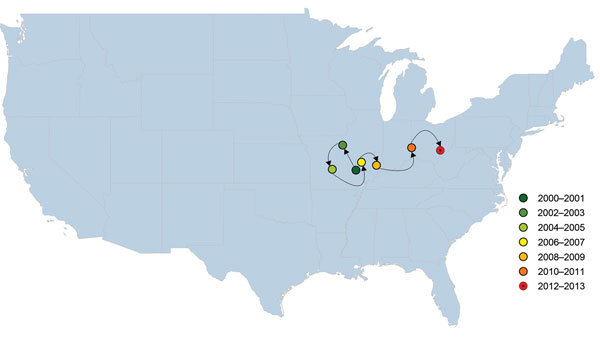
Weighted mean geographic center for proportions of methicillin-resistant *Staphylococcus aureus* (MRSA) USA300 strain type, United States, 2000–2013. This map shows the likely trend in the spread of USA300 as a proportion of all MRSA isolates that underwent genotyping, but the trajectory could be biased by large studies or lack of studies in certain states in specific years. The final mean center for 2012–2013 is represented differently to indicate that it is based on a small number of isolates.

The weighted mean geographic center of USA300 shifted from the start of the study period (2000–2001 studies) to the end of the study period (2012–2013 studies) (Figure 4). The general movement was from west to east; the early weight of USA300 isolates was heaviest in the Midwest, gradually moving toward the mid-Atlantic states during the study period. The mean center was pulled westward during 2002–2005 by studies performed in Alaska and Hawaii during those years.

**Figure 5:**
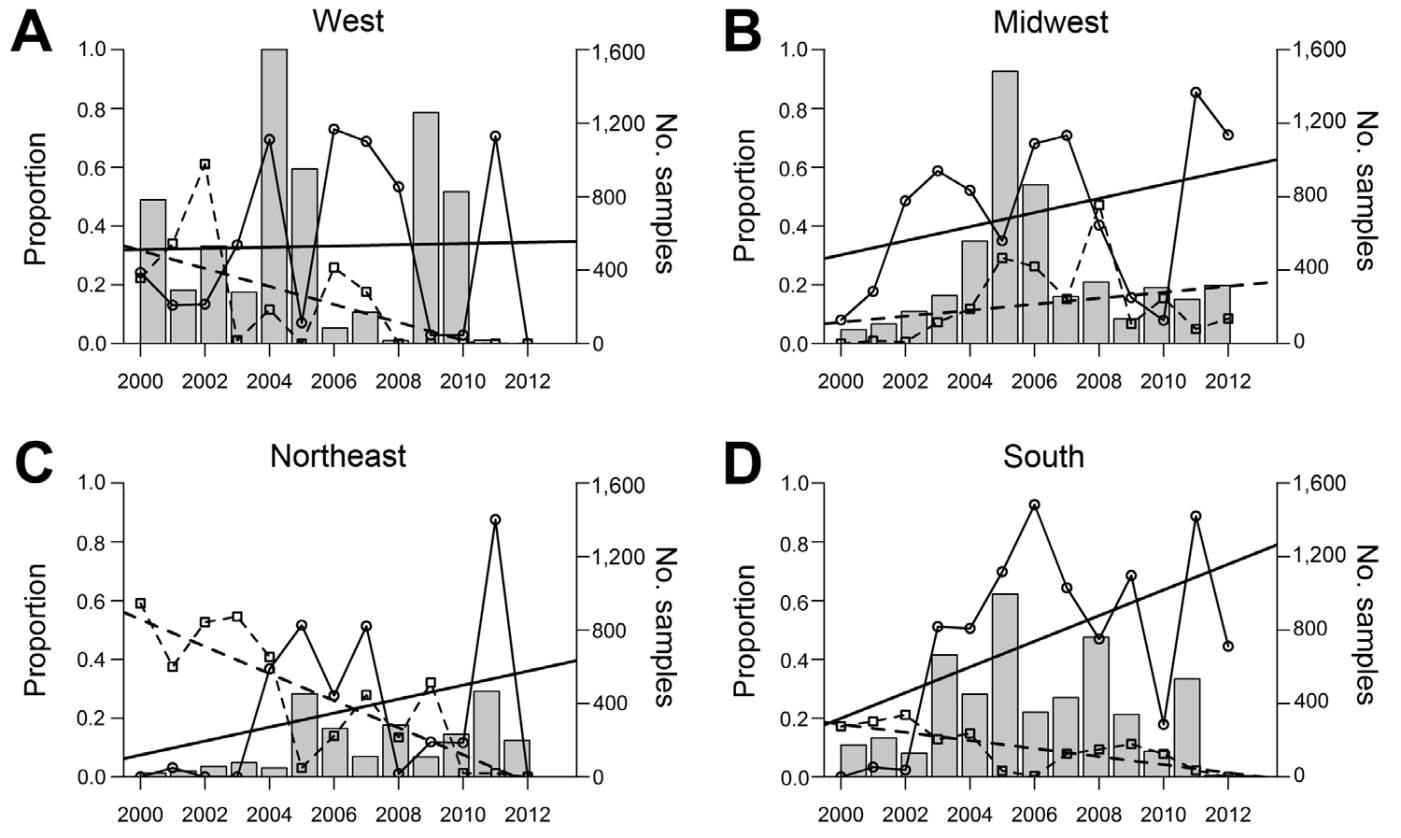
Proportion of methicillin-resistant *Staphylococcus aureus* USA300 and USA100 strain types and total sample size in 4 Census regions, United States 2000–2013. A) West. B) Midwest. C) Northeast. D) South. Linear regression lines are fit for each type. Solid line, USA300; dashed line, USA100.

When studies were assigned to 1 of 4 Census regions and the USA300 and USA100 proportions were charted over time (Figure 5), regional differences in the proportions of these 2 types differed by region. The number of isolates reported in each region also differed during the study. For the West, the general pattern over time was a series of peaks and troughs of USA300 reporting and a gradual decline in USA100. In the South and Northeast the general pattern over time was increasing USA300 proportions and decreasing USA100 proportions. The decrease in USA100 appeared particularly sharp in the Northeast. By contrast, in the Midwest, both USA300 and USA100 gradually increased, although the share of USA300 percentages was consistently higher. Cochran-Armitage tests for trend indicated statistically significant (p<0.0001) trends in USA300 and USA100 across all 4 Census areas.

## Discussion

We demonstrated that USA300 did not emerge simultaneously throughout the United States. It emerged earlier in the western part of the country and only later on the eastern seaboard. The published literature, used as a proxy for MRSA surveillance, suggests that USA300 appeared during 2000 in several states across the country, including California, Texas, and midwestern states. In subsequent years, USA300 constituted a large share of total reported MRSA isolates.

USA100, the predominant health care–associated MRSA strain type in the United States, in contrast, already constituted a larger proportion of reported MRSA isolates in the earlier years of the study in eastern US states. Over time, USA300 dominated among reported MRSA strain types in the Midwest and East. This finding is most clearly demonstrated by the focus of the geographic center of USA300 in Missouri/Illinois that gradually shifted toward the East during the latter years of the study.

Regionally, USA300 was present in higher proportions during the early years of the study in western states than other Census areas. Although Cochran-Armitage tests indicated statistically significant trends in all regions for both USA300 and USA100, trends toward an increase in the proportion of MRSA isolates that were USA300 were strongest in the Midwest and South, and large declines in USA100 proportions were observed in the Northeast, South, and West. These findings correspond to generally perceived but never formally tested hypotheses on the origins and spread of USA300 MRSA in the United States. The decline in the relative proportion of USA100 isolates reported during the study period most likely resulted from the increased attention to infection control in hospitals and a corresponding decrease in nosocomial health care–associated MRSA (*31*).

Our study is subject to several limitations. Most important, the data were not derived from a single sample of MRSA isolates collected prospectively or with equal representation of all states or regions. The data were derived instead from the extant literature in which authors chose to perform genotyping. Unlike an ideal prospective surveillance study that would include data selected to represent a sample of the entire population, we had available only published studies, which might have biased our results if in a given period. For example, if studies were more or less likely in 1 region of the country to focus on community-associated or health care–associated infections, if few studies were performed in a given region, or if smaller studies predominated in a given region compared with the rest of the country, our results could be biased. We attempted to correct for this lack of complete data by relying on all available data in each studied 2-year period to determine the geographic center of USA300 among MRSA isolates. The 2012–2013 center in particular was calculated from a small number of studies in the eastern portion of the country.

Second, although the articles included were identified through a rigorous literature review, additional published studies reporting a large number of isolates did not use any genotyping or did not use modalities of genotyping that met our inclusion criteria. Had these studies genotyped their reported isolates or genotyped them using methods that we chose to include, they might have altered the results of the study. Third, we eliminated from consideration in our analysis of geographic spread studies in which no US state of collection was named for studied MRSA isolates. This exclusion might have introduced error into our results, but we have no reason to believe that this error would systematically bias our findings because a specific region is unlikely to be overrepresented or underrepresented given this exclusion criterion. Fourth, although we made every attempt to exclude studies that reported previously published isolates, some isolates might been included more than once in our analyses. We identified specific strain names in the MRSA TypeCat whenever they were included in a published report to avoid repeated entries, but many authors did not identify specific isolate designations in their publications. Finally, many articles with genotyping information were not included because they provided inadequate typing data to identify USA300 or USA100 isolates by the criteria that we used to define these 2 strain types. Some articles, for example, identified isolates that bear the genetic determinants of PVL and SCC*mec* type IV, strongly suggesting a USA300 isolate, but we did not include these isolates in our analysis. We believe that the criteria that we chose for USA300 isolates, although arbitrary, were appropriately conservative to avoid misclassification of other community-associated MRSA strain types as USA300.

Our study examined the geographic distribution of USA300, a strain type of MRSA that emerged in the late 1990s to cause the fourth wave of resistance in *S. aureus* and came to predominate as a cause of MRSA infections in the United States during the course of approximately a decade. The reasons for the geographic pattern of emergence of USA300 from west to east are not yet known. However, this study represents an attempt to document the movement of a successful epidemic strain type of MRSA geographically over a prolonged period from its earliest emergence to predominance among MRSA strain types in a country. Our study might provide a model for understanding the emergence of a future, novel, fit strain type of antimicrobial drug–resistant *S. aureus* that could make up the fifth wave of resistance in *S. aureus* and is particularly relevant given increased focus and funding from the US government on developing a national strategy to combat antimicrobial drug–resistant bacteria (*32*). Such a strategy should include a national surveillance program that can detect the regional emergence of virulent new strains to inform local empiric therapy.

Technical Appendix Underlying data used to construct Figure 3.
